# Digital health readiness among rural hypertensive patients: a latent profile analysis

**DOI:** 10.3389/fpubh.2026.1786213

**Published:** 2026-04-15

**Authors:** Cuijuan Lin, Ershan Xu, Jiangnan He, Yingzi Tang, Ying Xiong, Yan Pu

**Affiliations:** 1 School of Nursing, Xiangtan Medicine & Health Vocational College, Xiangtan, China; 2 School of Nursing, Hunan University of Medicine, Huaihua, China; 3 Dongyang Town Health Center, Liuyang, China

**Keywords:** cardiovascular disease risk perception, digital health readiness, health ecological model, latent profile analysis, public health, rural hypertensive patients, social support

## Abstract

**Background:**

Driven by the rapid advancements in big data and artificial intelligence technologies, digital health tools have become deeply integrated into healthcare systems, offering novel pathways for blood pressure control and management. However, rural patients with hypertension face greater obstacles in accessing and utilizing digital technologies due to disparities in healthcare resources, education levels, and digital infrastructure. This study aims to identify latent classes of digital health readiness among rural hypertensive patients and explore their predictors based on the Health Ecological Model using latent profile analysis.

**Methods:**

This cross-sectional study followed the Strengthening the Reporting of Observational Studies in Epidemiology (STROBE) guidelines and collected data from 980 rural hypertensive patients across three townships in Hunan Province, China. Relevant factors were identified based on the Health Ecological Model. Research instruments included a General Information Questionnaire (23 items), the Social Support Rating Scale (10 items), the Cardiovascular Disease Risk Perception Assessment Tool (1 item), and the Digital Health Readiness Questionnaire (18 items). Latent profile analysis was employed to identify distinct subgroups of digital health readiness, and multivariate logistic regression analysis was used to determine predictive factors for each class.

**Results:**

Latent profile analysis revealed three distinct subtypes of digital health readiness among rural hypertensive patients: Low Digital Health Readiness group (*n =* 247, 25.2%), Moderate Digital Health Readiness group (*n =* 533, 54.4%), and High Digital Health Readiness group (*n =* 200, 20.4%). Multivariate logistic regression analysis demonstrated that age, duration of hypertension, number of chronic comorbidities, activities of daily living, regular exercise, awareness of “digital health,” cardiovascular disease risk perception, number of children, social support rating, educational level, employment status, physician recommendation to use information-based blood pressure management devices, and home wireless network coverage were significant factors influencing digital health readiness.

**Conclusion:**

These findings underscore the characteristics associated with lower digital health readiness. Rural healthcare institutions should develop tailored interventions targeting the specific vulnerabilities of different hypertensive patient populations and strengthen social support systems to enhance digital health readiness among rural patients with hypertension.

## Introduction

Hypertension is a major risk factor for cardiovascular disease (CVD), 245 million people in China are now affected, with rural prevalence already exceeding that in cities (1). Yet patients’ awareness (51.6%) and control rates (45.8%) are low, and only 16.8% receive treatment (2). Fueled by rapid advances in big data and artificial intelligence, digital-health technologies have been deeply integrated into medical care, offering a new route for blood-pressure control and management (3). At present, health-care organizations chiefly use social media (4, 5), health-management apps (6, 7), web platforms (8) and smart wearables (9) to support daily behavior management, continuous monitoring and online consultations for hypertensive patients. The use of these digital - health tools not only provides hypertensive patients with real - time insight into their blood - pressure status (10) but also, via data analytics, offers personalized advice on diet (11), exercise, and other lifestyle changes (12, 13). Clinicians can also remotely monitor patients’ blood - pressure trends and promptly modify treatment plans, enhancing therapeutic outcomes and reducing cardiovascular risk (14).

Despite these advantages, scaling and implementing digital health in hypertension care still poses challenges. Adoption and digital literacy differ across regions and socio - economic groups, potentially exacerbating inequities in access to digital - health services (15). This gap is especially pronounced between urban and rural China (16). Constrained by shortages of medical resources, lower education levels, and under - developed digital infrastructure, rural hypertensive patients face greater barriers to accessing and using digital health tools (17). To help them bridge the digital divide and better manage their hypertension through these technologies, a scientific assessment of their “digital - health readiness” is an essential first step. Only by accurately identifying the specific characteristics and predictors of readiness among rural hypertensive patients can targeted and actionable interventions be designed (18). In 2021, the World Health Organization defined digital health in the “Global Strategy on Digital Health (2020 - 2025)” (19) as the field of knowledge and practice associated with the development and utilization of digital technologies to improve health.

Although previous research has been conducted on hypertensive patients in the field of digital health, studies have primarily focused on digital health literacy, including scale development (20), multidimensional analyses of influencing factors (21, 22), and interventions (23, 24). Digital health literacy refers to an individual’s ability to search for, access, evaluate, and apply health information through digital technologies (25). However, digital health readiness is a multidimensional concept that builds upon digital health literacy by incorporating additional dimensions such as digital usage patterns, integrated digital skills, digital literacy, and willingness to learn (26). It represents a shift from the “can use” perspective of digital health literacy to a more comprehensive “willing and able to sustain usage” framework, enabling a more holistic assessment of preparedness for digital health interventions in routine clinical practice. Several scholars have developed instruments to assess digital health readiness (18, 27, 28). Xu e al. (16) translated and culturally adapted the Digital Health Readiness Questionnaire originally developed by Dr. Scherrenberg, subsequently validating its reliability and validity among hypertensive patients in rural China. This effort provides an empirically supported instrument for assessing digital health readiness within this specific rural population. Digital health readiness is likely to be influenced by the interaction of multiple factors. First, at the individual level, age and educational attainment are consistently significant predictors of digital-health readiness (29). Second, technology-specific anxiety and trust moderate the translation of predisposing characteristics into actual uptake (30, 31).

Third, socio-technical environmental conditions shape readiness at the organisational and community levels (32, 33). Finally, intervention design must accommodate cultural and linguistic diversity (34). However, there is currently a lack of adequate research on the status quo and determinants of digital health readiness in China. Meanwhile, the extant literature on determinants of digital-health readiness has overwhelmingly treated the target population as a homogeneous entity, neglecting to identify and characterise distinct readiness profiles within the sample. This “one-size-fits-all” assumption risks attenuating the ecological validity of findings, because divergent technology-acceptance needs and barriers that operate across readiness strata remain concealed.

Latent Profile Analysis (LPA) offers a person-centred alternative: it posits that an unobserved categorical latent variable generates differential distributions of observed readiness indicators, enabling the empirical extraction of mutually exclusive subgroups that are internally homogeneous yet externally heterogeneous (35, 36). Applying LPA to digital-health readiness can therefore uncover qualitatively different “readiness phenotypes.” Subsequent interrogation of factor–profile associations will elucidate whether—and how—predictors vary across latent classes, thereby furnishing an evidence base for precision interventions that are tailored to the unique configuration of barriers and facilitators characterising each readiness segment.

**Figure 1:**
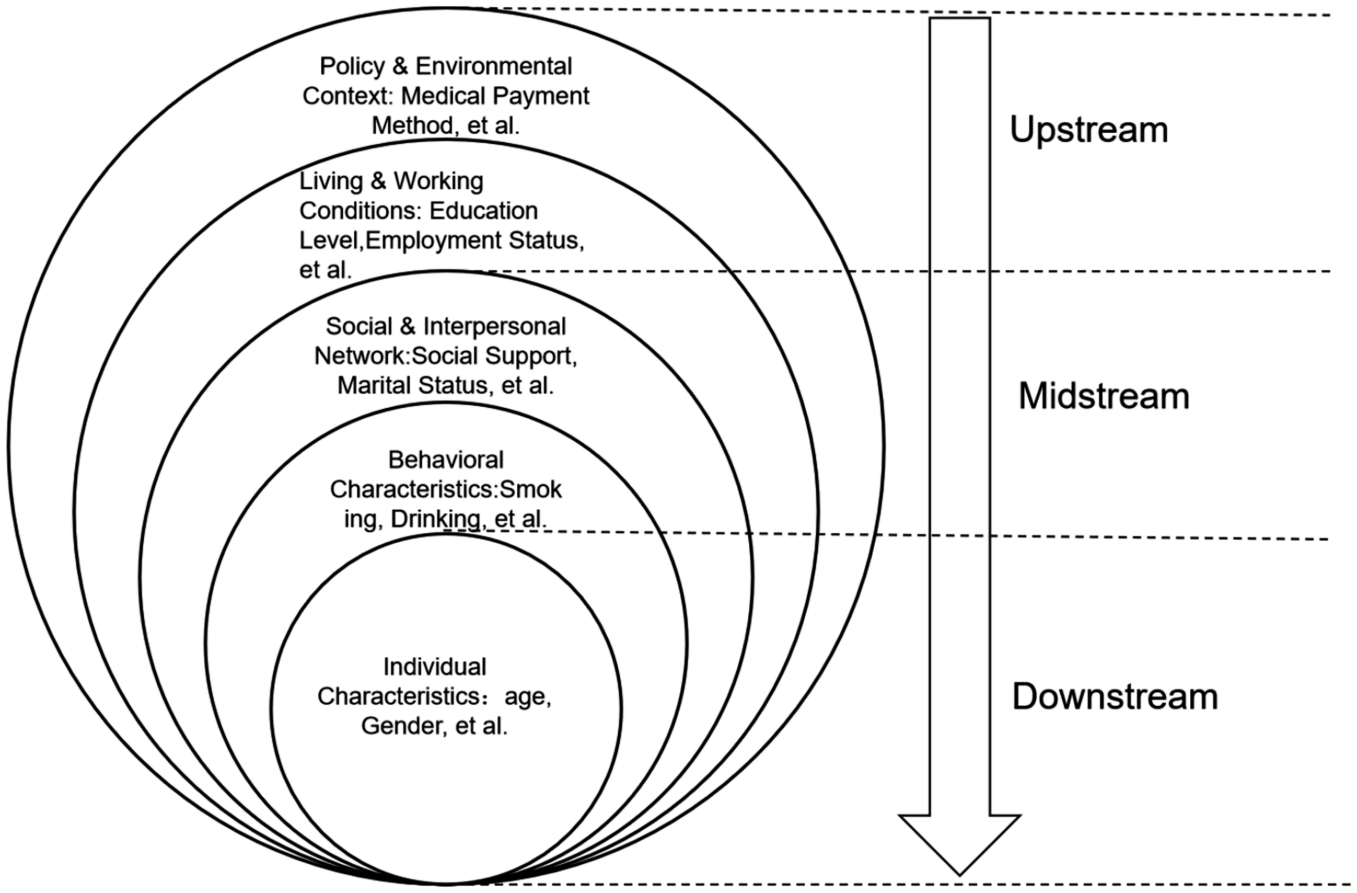
Health ecology model frame.

The Health Ecological Model (HEM) posits that health is the product of the joint influence of individual factors, lifestyle behaviors, health - care services, social environments, and the natural environment. These influences are conceptualized across five hierarchical levels from the inside out: Individual Characteristics, Behavioral Characteristics, Social and Interpersonal Network, Living and Working Conditions, Policy and Environmental Context (37, 38) (see Figure 1 for the theoretical model). Digital health readiness—a key prerequisite for hypertensive patients to adopt digital blood - pressure management technologies—encompasses both personal cognitions and context - dependent characteristics, thereby aligning well with the multi - level logic of HEM. Consequently, this study adopts the Health Ecological Model as its theoretical framework to develop a Digital Health Readiness Model for Rural Hypertensive Patients. Using latent profile analysis, we identify distinct typologies of digital health readiness among rural hypertensive patients and examine their predictive factors. This study aims to precisely identify barriers to digital health engagement and inform the design of targeted, differentiated intervention strategies, thereby enhancing the sustainability and equity of digital blood pressure management in rural settings.

## Methods

### Design

A multicenter, cross-sectional study was conducted from September to October 2025. To ensure comprehensive and accurate reporting of the research, the Strengthening the Reporting of Observational Studies in Epidemiology (STROBE) guidelines (39) were followed (see Supplementary File 1).

### Participants, setting and sampling

We employed a convenience sampling strategy to recruit hypertensive patients registered in the chronic disease management systems of three rural township health centers across Hunan Province, China. These centers were purposively selected to represent diverse socioeconomic gradients: Liuyang (economically developed county, 3 villages), Zhuzhou (moderately developed city, 4 villages), and Loudi (less developed region, 3 villages), encompassing 10 villages in total. Eligible participants were identified through electronic medical records and community health records, ensuring comprehensive coverage of the target population within selected villages.

Inclusion criteria: Meet the diagnostic criteria of the hypertension guidelines; Be conscious, capable of reading or expressing themselves verbally, and have no communication barriers; Be informed and voluntarily participate in this study.

Exclusion Criteria: Suffer from severe physical and mental illnesses; Be unable to cooperate due to hearing impairment, language disorders, or other issues; Have secondary hypertension.

### Data collection procedures and instruments

Data collection commenced following the acquisition of administrative authorizations from three township health centers. At each site, two registered nurses screened potential participants from electronic health management databases according to the predefined inclusion and exclusion criteria and subsequently informed eligible hypertensive patients regarding the home-based survey visits. A team of 50 nursing students was deployed to conduct face-to-face interviews in participants’ residences. Prior to fieldwork initiation, all research assistants completed a standardized two-day training program encompassing: (a) study objectives, protocol adherence, and ethical considerations; (b) standardized administration procedures using the Wenjuanxing electronic data capture platform; (c) scripted explanations for potentially ambiguous constructs (e.g., operational definitions of “digital health”); and (d) techniques to mitigate social desirability bias, including assurance of response anonymity and non-judgmental interviewing approaches. Upon arrival at participants’ residences, nursing students provided comprehensive information regarding the study purpose and procedures, after which written informed consent was obtained. Data collection was facilitated through the Wenjuanxing mobile application, with participants completing questionnaires independently or with minimal assistance as required. Mandatory completion of all scale items was enforced by the electronic system prior to final submission. No personally identifiable information was collected by the investigators. Participants had the right to withdraw from the study at any time. The systematic exclusion of invalid questionnaires based on the following criteria: (a) logical inconsistencies between related items; (b) completion times below 3 min or exceeding 30 min without documented justification; and (c) response patterns indicative of careless responding, including straight-lining or systematic zigzag patterns.

Following a comprehensive literature review and deliberations by the collaborative research team (40–42), this study identified 25 variables based on the Health Ecological Model and stratified them across five ecological levels as determinants of digital health readiness among rural hypertensive patients. (1) Individual Characteristics: Age, Gender, BMI, Duration of Hypertension, Family History of Hypertension, Number of Chronic Comorbidities, Activities of Daily Living, Number of Hospitalizations. (2) Behavioral Characteristics: Smoking, Drinking, Taking B Vitamins, Taking Folic Acid Tablets, Regular Exercise, Awareness of “Digital Health,” Cardiovascular Disease Risk Perception. (3) Social and Interpersonal Network: Social Support, Marital Status, Number of Children. (4) Living and Working Conditions: Education Level, Employment Status, *Per Capita* Monthly Household Income, Doctor - recommended Use of digital - based Blood Pressure Management Devices, Main Medical Institution for Visits, Home Wireless Network Coverage. (5) Policy and Environmental Context: Medical Payment Method.

The data collection instrument included a 52 - item self - reported questionnaire, which consisted of General Information (23 items), Social Support Rating Scale (10 items), Cardiovascular Disease Risk Perception Assessment Tool (1 item), and Digital Health Readiness Questionnaire (18 items).

- Social Support Rating Scale (SSRS):

The SSRS is used to assess the level of social support an individual has obtained. It was compiled by Xiao (43) and consists of three dimensions: objective support, subjective support, and utilization of support, with a total of 10 items. For items 1 to 4 and 8 to 10, selecting options 1 to 4 are scored as 1 to 4 points, respectively. In item 5, the four sub-items are scored on a four-point scale (1 = none, 4 = full support), and the sum of the scores of the four sub - items is the score for item 5. For items 6 and 7, selecting “no sources” is scored as 0 points, while selecting “having sources” means scoring according to the number of sources. The total score is the sum of the scores of the 10 items. The higher the total score, the higher the level of social support. The total score ranges from 12 to 66 points. The higher the score, the higher the level of social support. A total score of ≤ 22 indicates a low level of social support, 23–44 indicates a moderate level, and 45–66 indicates a high level. The Cronbach’s Alpha coefficient of the SSRS is 0.90.

II Cardiovascular Disease Risk Perception Assessment Tool

The single-item cardiovascular disease risk perception assessment tool was used to measure the absolute risk perception level of the subjects (44). The assessment item is “How do you perceive your risk of developing cardiovascular disease in the next 10 years?” The risk level is indicated on a scale of 0 to 10, where 0 means absolutely impossible and 10 means absolutely certain. Referring to the pain assessment grading in this study, the levels 0–3 are defined as low risk, 4–6 as moderate risk, and 7–10 as high risk (45).

III Digital Health Readiness Questionnaire (DHRQ)

Developed by Dr. Scherrenberg et al. (28), and translated into Chinese by Xu et al. (16), this questionnaire aims to assess patients’ mastery of digital health knowledge and their willingness to learn new digital health tools in the future. The questionnaire consists of five subscales (namely digital use ability, digital skills, digital literacy, digital health literacy, and digital learning ability), with a total of 18 items scored on a 1–5 Likert scale. The higher the score, the stronger the user’s mastery of digital health knowledge and tools, and their willingness to learn. Confirmatory factor analysis (CFA) was conducted to evaluate the construct validity of the Digital Health Readiness Questionnaire (DHRQ). The results indicated an acceptable model fit: *χ*^2^/df = 9.761, CFI = 0.943, TLI = 0.920, SRMR = 0.046, and RMSEA = 0.095. Standardized factor loadings ranged from 0.704 to 0.934. These fit indices were largely consistent with those reported in Xu’s validation study (16) (*χ*^2^/df = 4.897, CFI = 0.914, TLI = 0.895, RMSEA = 0.109, SRMR = 0.0765). In this study, with an overall Cronbach’s *α* coefficient of 0.960. Subscale analyses yielded the following *α* values: Digital Usability (*α* = 0.872), Digital Skills (*α* = 0.904), Digital Literacy (*α* = 0.850), Digital Health Literacy (*α* = 0.911), and Digital Learnability (*α* = 0.936). All subscale coefficients exceeded the recommended threshold of 0.70, indicating robust reliability across all dimensions of the instrument.

### Samples

Methodological guidelines for Latent Profile Analysis (LPA) indicate that *N* ≥ 300 represents the minimum threshold for ensuring basic stability of parameter estimates, whereas *N* ≥ 500 is recommended for exploratory analyses aiming to identify between two and four latent classes (46). Ultimately, 1,105 questionnaires were distributed, yielding 980 valid responses and an effective response rate of 88.69%. The 125 invalid questionnaires included the following: logical inconsistencies (*n =* 40), completion times of <3 min or more than 30 min without a valid explanation (*n =* 57), and patterned response tendencies (*n =* 28). This sample size exceeds recommended thresholds and is therefore considered adequate for robust LPA estimation.

### Data analysis

LPA was implemented via M-plus 8.3 software to construct latent profile models of digital health readiness among rural hypertensive patients, with model fit indices guiding determination of the optimal class number (47). Evaluative metrics incorporated log-likelihood (LL), Akaike Information Criterion (AIC), Bayesian Information Criterion (BIC), adjusted BIC (aBIC), entropy coefficient, Lo–Mendell–Rubin adjusted likelihood ratio test (LMRT), and bootstrapped likelihood ratio test (BLRT). Model selection criteria were defined as follows: superior fit is indicated by lower AIC, BIC, and aBIC values coupled with higher entropy; LMRT and BLRT with significant *p*-values (*p* < 0.05) suggest the k-class model is statistically preferable to the k-1 class alternative (48). Based on these parameters, the optimal fitting model was selected to categorize rural hypertensive patients into discrete digital health readiness profiles. The theoretical underpinnings of LPA posit that response distributions across observed variables can be attributed to a finite set of mutually exclusive latent classes, each characterized by distinct response patterns on the measured indicators (49).

The SPSS 26.0 software was utilized to conduct statistical analysis. Frequency and percentage were used to describe count data, whereas mean and standard deviation characterized measurement data. The chi-square test was applied to identify differences in digital health readiness among rural hypertensive patients based on general information characteristics and cardiovascular disease risk perception. Spearman correlation analysis examined the relationships between digital health readiness and social support rating. Comparisons among different digital health readiness groups were made using one-way ANOVA, and post-hoc comparisons were conducted using LSD-t tests. When the variances are not equal (*p* < 0.05), the Kruskal-Wallis test is used.

Multivariate logistic regression analysis was used for univariate and multivariate analyses of the potential categories of digital health readiness among rural hypertensive patients. Variables that were statistically significant in univariate analysis (*p* < 0.01) were used as independent variables, with the results of the latent class analysis serving as the dependent variable. A two-sided *p*-value of less than 0.05 was considered statistically significant.

## Results

### Common method Bias test

Common method variance was assessed using Harman’s single - factor test. Exploratory factor analysis without rotation extracted 14 factors with eigenvalues >1. The first factor explained 24.765% of the variance, well below the 40% threshold. These results indicate that common method bias was unlikely to confound findings.

### Sample characteristics

We analyzed data from 980 rural hypertensive patients in Hunan province who valid completed the survey. Among the survey participants, those aged 60 years or older accounted for 57.46%, and females accounted for 50.68%. Detailed characteristics are shown in Table 1. The total scores of social support rating, digital health readiness of rural hypertensive patients were (37.58 ± 6.32) and (53.03 ± 16.42). The scores for each dimension of social support rating were objective support (8.74 ± 2.71), subjective support (21.29 ± 3.67), utilization of support (7.55 ± 1.91). The scores for each dimension of digital health readiness were digital usability (6.61 ± 2.61), digital skills (15.89 ± 5.58), digital literacy (8.10 ± 2.91), digital health literacy (8.20 ± 3.04), digital learnability (14.22 ± 4.67).

**Table 1:** Demographic characteristics and relevant variable differences in digital health readiness.

Variables	*n*	Percentage (%)	*p*
Age (years)			
≤49	186	19.64%	<0.001
50–59	218	21.27%	
60–69	295	29.86%	
≥70	281	27.60%	
Gender			
Male	493	49.32%	<0.001
Female	487	50.68%	
Marital status			
Married	823	84.07%	<0.001
Unmarried or Divorced	21	2.90%	
Widowed	136	13.03%	
Number of children			
0–1	111	11.23%	<0.001
2–3	765	78.64%	
≥4	104	10.14%	
Education level			
Primary School and Below	476	48.42%	<0.001
Junior High School	310	31.49%	
Senior High School/Vocational School	143	14.66%	
Junior College	27	2.90%	
Bachelor’s Degree and Above	24	2.53%	
Employment status			
Employed	238	24.71%	<0.001
Unemployed	470	47.60%	
Retired	272	27.69%	
*Per Capita* Monthly Household Income (Yuan/month)			
≤1,000	136	14.12%	<0.001
1,001–2000	188	18.73%	
2001–3,000	245	25.52%	
3,001–4,000	216	22.35%	
≥4,000	195	19.28%	
Medical payment method			
Urban and Rural Resident Medical Insurance	716	72.22%	<0.001
Employee Medical Insurance	77	8.51%	
Self-Payment	159	16.38%	
Other	28	2.90%	
Smoking			
Yes	301	30.59%	0.098
No	679	69.41%	
Drinking			
No	508	51.84%	0.003
Occasionally	369	37.65%	
Often	103	10.51%	
Taking B Vitamins			
Yes	186	18.98%	0.058
No	794	81.02%	
Taking folic acid tablets			
Yes	148	15.10%	0.003
No	832	84.90%	
BMI			
Underweight	70	7.14%	<0.001
Normal	459	46.84%	
Overweight	312	31.84%	
Obesity	139	14.18%	
Regular exercise			
Yes	304	31.02%	<0.001
No	676	68.98%	
Duration of hypertension (years):			
≥10	546	55.71%	<0.001
5–10	315	32.14%	
≤5	119	12.14%	
Family history of hypertension			
Yes	255	26.02%	<0.001
No	725	73.98%	
Number of chronic comorbidities			
None	553	56.43%	<0.001
1 Type	284	28.98%	
≥2 Types	143	14.59%	
Activities of daily living			
Normal	640	65.31%	<0.001
Decreased to Different Extents	299	30.51%	
Significant Barriers	41	4.18%	
Number of Hospitalizations (in the past five years)			
None	356	36.33%	<0.001
1–2 Times	472	48.16%	
≥3 Times	152	15.51%	
Awareness of “Digital Health”			
Aware	91	9.29%	<0.001
Know a Little	405	41.33%	
Completely Unaware	484	49.39%	
Doctor - recommended use of digital - based blood pressure management devices			
Yes	365	37.24%	<0.001
No	615	62.76%	
Main medical institution for visits			
Tertiary*	303	30.92%	<0.001
Secondary**	244	24.90%	
Community Hospital***	433	44.18%	
Home wireless network coverage			
Yes	882	90.00%	<0.001
No	98	10.00%	
Cardiovascular disease risk perception			
Low risk	472	48.16%	0.014
Moderate risk	360	36.73%	
High risk	148	15.10%	

*Tertiary Hospital: Regional medical centers (> 500 beds) that provide comprehensive/specialized care, teaching, and research. **Secondary Hospital: District-level hospitals (101 to 500 beds) that handle complex cases and regional referrals. ***Community Hospital: Primary care institutions (< 100 beds) focused on preventive care and common illnesses.

### Correlational analysis of social support rating and digital health readiness

The results of Spearman correlation analysis indicated that the total score and the scores on each dimension of the social support rating and digital health readiness were positively correlated (both *p* < 0.01) (Table 2).

**Table 2:** Spearman correlation analysis between the variables of social support rating and digital health readiness (r).

**Variable**	**Objective support**	**Subjective support**	**Utilization of support**	**SSRS**
Digital skills	0.189^**^	0.219^**^	0.104^**^	0.242^**^
Digital literacy	0.179^**^	0.233^**^	0.125^**^	0.252^**^
Digital health literacy	0.201^**^	0.241^**^	0.131^**^	0.273^**^
Digital learnability	0.187^**^	0.254^**^	0.162^**^	0.283^**^
Digital usability	0.184^**^	0.228^**^	0.113^**^	0.248^**^
Dhrq	0.215^**^	0.271^**^	0.140^**^	0.296^**^

**p < 0.01.

### Latent profile analysis of digital health readiness

**Figure 2:**
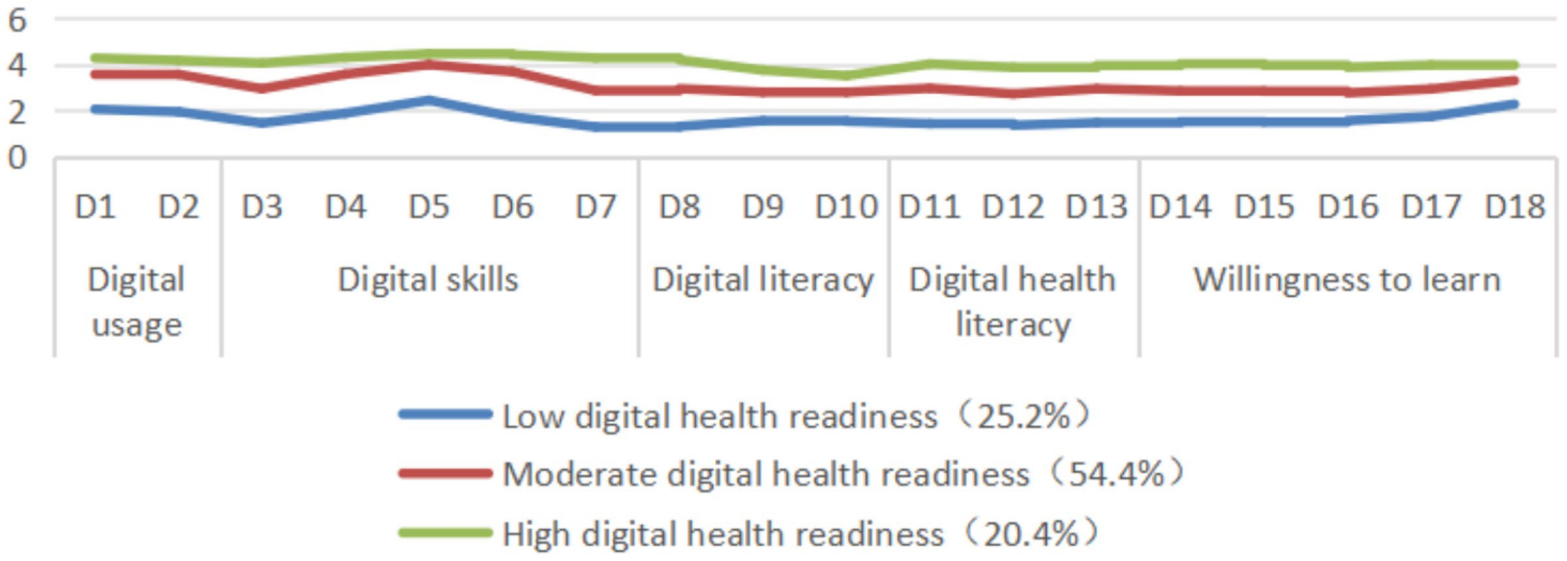
Varying profiles of digital health readiness among rural hypertensive patients.

Considering both model fit indices and class interpretability, the three - class model is the optimal solution. As shown in Table 3, first, the three - class model reached significant levels in both the LMRT and BLRT tests (*p* < 0.001), indicating that it is significantly better than the two - class model. Meanwhile, the Entropy value was as high as 0.958, indicating extremely high classification accuracy. Second, the group size distribution of the three - class model is reasonable (25.2, 54.4, 20.4%), with no overly small classes, ensuring the stability and generalizability of each class. Moreover, compared with the four - class and five - class models, the three - class model has significantly decreased and stabilized in information criteria (AIC, BIC, aBIC), and has avoided the complexity and interpretability difficulties brought by higher - class models. Therefore, the three - class model has achieved the best balance between statistical rigor and practical interpretability, providing a clear and robust basis for subsequent population characteristic analysis and intervention strategy formulation. The score distribution of the latent types of digital health readiness on the 18 items of the DHRQ scale is shown in Figure 2. Type 1, characterized by universally low scores on the five dimensions of digital health readiness, accounted for 25.2% (247/980) of the total number of participants and was named the Low Digital Health Readiness group. Type 2, characterized by universally moderate scores on the five dimensions of digital health readiness, accounted for 54.4% (533/980) of the total number of participants and was named the Moderate Digital Health Readiness group. Type 3 had the highest scores on all items, accounting for 20.4% (200/980) of the total number of participants, and was named the High Digital Health Readiness group.

**Table 3:** Latent profile analysis of digital health readiness with model fit results (*n =* 980).

Profile	AIC	BIC	aBIC	LMRT (*p*-value)	BLRT (*p*-value)	Entropy	Group size for each profile (ratio)				
							1	2	3	4	5
1	55569.220	55745.172	55630.836								
2	47181.070	47449.885	47275.205	<0.001	<0.001	0.975	302 (0.308)	678 (0.692)			
3	44427.695	44789.374	44554.349	<0.001	<0.001	0.958	247 (0.252)	533 (0.544)	200 (0.204)		
4	43063.378	43517.920	43222.551	0.0879	<0.001	0.958	141 (0.144)	464 (0.473)	189 (0.193)	186 (0.190)	
5	42303.946	42851.352	42495.638	0.0493	<0.001	0.925	143 (0.146)	172 (0.176)	249 (0.254)	246 (0.251)	170 (0.17)

The average probability of attribution for the three groups ranged from 97.7 to 99.0% (Table 4), indicating that the LPA classification results in this study are credible. The differences in total scores and scores on each dimension of the digital health readiness scale among the three groups were statistically significant (*p* < 0.001 for all comparisons). Importantly, the High Digital Health Readiness group had the highest total scores and scores on each dimension, as shown in Table 5.

**Table 4:** Average probability of attribution for each potential profile.

Class	Profile 1 (%)	Profile 2 (%)	Profile 3 (%)
1	0.990	0.010	0.000
2	0.008	0.979	0.013
3	0.000	0.023	0.977

**Table 5:** The total digital health readiness score and each dimension score of the 3 potential categories [score, $\bar{x}$ ± s (95% CI)].

Category	The total score	Digital usage	Digital skills	Digital literacy	Digital health literacy	Willingness to learn
Low digital health readiness	30.03 ± 8.23	4.00 ± 2.19	8.81 ± 3.46	4.40 ± 1.76	4.26 ± 1.70	8.56 ± 3.42
Moderate digital health readiness	56.09 ± 6.39 (24.81–27.31)^*^	7.14 ± 2.15 (2.82–3.45)^*^	17.06 ± 3.52 (7.76–8.77)^*^	8.54 ± 1.42 (3.90–4.39)^*^	8.64 ± 1.48 (4.14–4.61)^*^	14.70 ± 2.25 (5.75–6.54)^*^
High digital health readiness	73.26 ± 6.37 (16.05–18.30) ^#^	8.44 ± 1.65 (0.97–1.64)^#^	21.52 ± 2.67 (3.91–5.00)^#^	11.49 ± 1.83 (2.69–3.21)^#^	11.89 ± 1.62 (2.95–3.56)^#^	19.91 ± 2.52 (4.78–5.64)^#^
Kruskal-Wallis H test */p*	781.56/<0.001	341.45/<0.001	595.74/<0.001	663.43/<0.001	702.37/<0.001	652.16/<0.001

*Compared with the Low digital health readiness group; ^#^compared with the Moderate digital health readiness group; All 95% Confidence Intervals (CI) exclude zero, indicating robust differences between profiles.

### Univariate and multivariate logistic regression results for predicting external features on the 3-class pattern of digital health readiness

Variables showing statistical significance (*p* < 0.01) in the univariate analysis were entered into the multivariate analysis. The statistically significant predictors that differentiate each group are Age, Duration of hypertension, Number of chronic comorbidities, Activities of daily living, Regular exercise, Awareness of “Digital Health,” Cardiovascular disease risk perception, Number of children, Social support rating, Educational level, Employment status, Doctor - recommended use of digital - based blood pressure management devices, Home wireless network coverage (see Table 6).

**Table 6:** Univariate and multivariate logistic regression results for predicting external features on the 3-class pattern.

Variables	Univariate analysis				Multivariate analysis			
	Class 2 vs. Class 1 OR (95%CI)	*p*	Class 3 vs. Class 1 OR (95%CI)	*p*	Class 2 vs. Class 1 OR (95%CI)	*p*	Class 3 vs. Class 1 OR (95%CI)	*p*
Individual characteristics								
Age (years)								
(≥70 as ref) ≤49 50–59 60–69	20.781 (8.196–52.690) 5.653 (3.460–9.233) 2.090 (1.463–2.987)	<0.001 <0.001 <0.001	107.730 (38.918–298.211) 15.162 (7.799–29.478) 3.325 (1.828–6.049)	<0.001 <0.001 <0.001	5.322 (1.781–15.909) 2.078 (1.091–3.957) 1.237 (0.792–1.932)	**0.003** **0.026** 0.350	22.703 (6.189–83.283) 4.509 (1.789–11.312) 2.265 (1.071–4.790)	**<0.001** **0.001** **0.032**
Gender: (Female as ref) Male	1.229 (0.907–1.666)	0.183	2.525 (1.718–3.713)	<0.001	0.954 (0.638–1.426)	0.818	1.582 (0.914–2.738)	0.101
BMI: (Obesity as ref) Underweight Normal Overweight	0.579 (0.301–1.114) 0.990 (0.630–1.554) 1.805 (1.095–2.974)	0.102 0.964 0.021	0.575 (0.254–1.299) 0.786 (0.454–1.363) 1.569 (0.866–2.843)	0.183 0.392 0.137				
Duration of Hypertension (years): (≤5 as ref) ≥10 5–10	2.537 (1.636–3.936) 1.908 (1.205–3.023)	<0.001 0.006	4.815 (2.491–9.306) 2.372 (1.178–4.775)	<0.001 0.016	1.418 (0.805–2.500) 1.819 (1.029–3.214)	0.227 **0.039**	1.530 (0.622–3.764) 2.053 (0.817–5.156)	0.354 0.126
Family History of Hypertension: (No as ref) Yes	1.564 (1.082–2.260)	0.017	1.819 (1.177–2.813)	0.007	1.261 (0.786–2.024)	0.335	1.179 (0.683–2.179)	0.600
Number of Chronic Comorbidities: (≥2 Types as ref) None 1 Type	2.264 (1.504–3.408) 1.211 (0.786–1.868)	<0.001 0.386	8.631 (4.085–18.235) 3.742 (1.712–8.182)	<0.001 0.001	1.227 (0.673–2.234) 0.937 (0.525–1.671)	0.504 0.826	3.092 (1.069–8.947) 2.468 (0.882–6.904)	**0.037** 0.085
Activities of Daily Living: (Significant Barriers as ref) Normal Decreased to Different Extents	6.423 (3.158–13.066) 3.914 (1.896–8.080)	<0.001 <0.001	17.637 (4.116–75.583) 5.063 (1.145–22.385)	<0.001 0.032	3.079 (1.265–7.494) 2.576 (1.080–6.144)	**0.013** **0.033**	2.593 (0.448–15.011) 1.378 (0.240–7.905)	0.288 0.719
Number of Hospitalizations (in the past five years): (≥3 Times as ref) None 1–2 Times	2.159 (1.390–3.355) 1.459 (0.977–2.180)	0.001 0.065	5.495 (2.867–10.534) 2.766 (1.474–5.192)	<0.001 0.002	0.938 (0.506–1.739) 0.758 (0.447–1.285)	0.839 0.303	0.938 (0.362–2.428) 0.644 (0.270–1.537)	0.894 0.321
**Behavioral Characteristics**								
Smoking: (No as ref) Yes	0.915 (0.658–1.273)	0.597	1.347 (0.907–1.999)	0.139				
Drinking: (Often as ref) No Occasionally	0.823 (0.485–1.397) 1.123 (0.646–1.953)	0.470 0.681	0.538 (0.291–0.996) 0.873 (0.462–1.649)	0.048 0.676				
Taking B Vitamins: (No as ref) Yes	1.286 (0.855–1.934)	0.227	1.690 (1.050–2.719)	0.031				
Taking Folic Acid Tablets: (No as ref) Yes	1.439 (0.904–2.291)	0.125	2.101 (1.241–3.558)	0.006	1.288 (0.737–2.254)	0.374	1.835 (0.902–3.733)	0.094
Regular exercise (No as ref) Yes	1.685 (1.182–2.402)	0.004	2.597 (1.716–3.928)	<0.001	1.471 (0.958–2.258)	0.078	2.410 (1.375–4.223)	**0.002**
Awareness of “Digital Health”: (Completely Unaware as ref) Aware Know a Little	2.505 (1.221–5.141) 2.088 (1.494–2.919)	0.012 <0.001	17.527 (8.097–37.940) 7.708 (4.880–12.175)	<0.001 <0.001	0.811 (0.328–2.006) 1.320 (0.871–1.999)	0.650 0.190	2.077 (0.739–5.840) 3.406 (1.914–6.062)	0.166 **<0.001**
Cardiovascular Disease Risk Perception: (high risk as ref) low risk moderate risk	2.709 (1.764–4.159) 1.840 (1.199–2.824)	<0.001 0.005	2.933 (1.712–5.025) 1.272 (0.722–2.238)	<0.001 0.405	1.236 (0.683–2.239) 0.992 (0.567–1.736)	0.484 0.979	0.557 (0.243–1.274) 0.427 (0.187–0.974)	0.166 **0.043**
**Social and Interpersonal Network**								
Marital Status: (Widowed as ref) Married Unmarried or Divorced	2.258 (1.530–3.332) 3.030 (0.796–11.535)	<0.001 0.104	5.935 (2.947–11.953) 16.000 (3.620–70.725)	<0.001 <0.001	0.717 (0.426–1.209) 0.991 (0.187–5.263)	0.212 0.991	0.539 (0.212–1.370) 2.373 (0.325–17.345)	0.194 0.394
Number of Children: (≥4 as ref) 0 or 1 2–3	4.528 (2.344–8.746) 3.530 (2.258–5.517)	<0.001 <0.001	8.974 (3.710–21.705) 5.055 (2.492–10.255)	<0.001 <0.001	2.483 (1.035–5.956) 3.135 (1.768–5.557)	**0.042** **<0.001**	1.604 (0.462–5.566) 3.038 (1.188–7.768)	0.456 **0.020**
Social Support Rating					1.069 (1.035–1.105)	**<0.001**	1.118 (1.069–1.170)	**<0.001**
**Living and Working Conditions**								
Education Level: (Bachelor’s Degree and Above as ref) Primary School and Below Junior High School Senior High School/Vocational School Junior College	0.124 (0.016–0.981) 0.446 (0.056–3.585) 1.100 (0.124–9.724) 0.800 (0.043–14.886)	0.048 0.448 0.932 0.881	0.014 (0.002–0.111) 0.161 (0.020–1.276) 0.452 (0.052–3.949) 1.385 (0.079–24.229)	<0.001 0.084 0.473 0.824	0.250 (0.027–2.318) 0.507 (0.054–4.775) 1.025 (0.101–10.446) 0.411 (0.019–8.782)	0.223 0.553 0.983 0.569	0.073 (0.007–0.773) 0.283 (0.027–2.984) 0.491 (0.043–5.626) 0.903 (0.040–20.556)	**0.030** 0.294 0.568 0.949
Employment Status: (Retired as ref) Employed Unemployed	9.490 (4.230–21.291) 0.766 (0.546–1.073)	<0.001 0.121	25.929 (11.040–60.893) 0.664 (0.411–1.072)	<0.001 0.094	3.621 (1.348–9.728) 1.161 (0.751–1.794)	**0.011** 0.503	3.958 (1.278–12.256) 1.282 (0.663–2.480)	**0.017** 0.460
*Per Capita* Monthly Household Income (Yuan/month) (≥4,000 as ref) ≤1,000 1,001–2000 2001–3,000 3,001–4,000	0.279 (0.164–0.474) 0.662 (0.396–1.107) 0.979 (0.591–1.622) 1.140 (0.666–1.949)	<0.001 0.116 0.935 0.633	0.080 (0.037–0.171) 0.278 (0.151–0.512) 0.387 (0.216–0.693) 0.684 (0.379–1.236)	<0.001 <0.001 0.001 0.208	0.604 (0.310–1.177) 1.167 (0.618–2.206) 1.158 (0.627–2.137) 1.487 (0.770–2.870)	0.138 0.634 0.640 0.237	0.374 (0.135–1.040) 1.045 (0.454–2.408) 0.749 (0.345–1.629) 1.242 (0.555–2.778)	0.059 0.917 0.467 0.598
Doctor - recommended Use of digital - based Blood Pressure Management Devices: (No as ref) Yes	2.861 (1.976–4.141)	<0.001	6.504 (4.228–10.003)	<0.001	1.745 (1.108–2.747)	**0.016**	2.762 (1.559–4.893)	**<0.001**
Main Medical Institution for Visits: (Community Hospital as ref) Tertiary Secondary	1.459 (1.013–2.100) 2.212 (1.488–3.290)	0.042 <0.001	3.989 (2.560–6.217) 2.684 (1.601–4.500)	<0.001 <0.001	0.998 (0.625–1.595) 1.510 (0.930–2.453)	0.995 0.096	1.726 (0.923–3.226) 1.653 (0.848–3.223)	0.087 0.140
Home Wireless Network Coverage: (No as ref) Yes	3.611 (2.274–5.734)	<0.001	3.651 (1.921–6.939)	<0.001	2.260 (1.278–3.997)	**0.005**	1.445 (0.593–3.522)	0.418
Policy and environmental context								
Medical Payment Method: (Other as ref) Urban and Rural Resident Medical Employee Medical Insurance Self-Payment	0.895 (0.362–2.213) 1.969 (0.609–6.362) 0.965 (0.367–2.533)	0.811 0.258 0.942	0.928 (0.288–2.989) 5.775 (1.448–23.032) 1.221 (0.354–4.202)	0.901 0.013 0.752				

OR, odds ratio; CI, 95% confidence interval. The bold values indicate that the corresponding statistical tests yielded *p* values <0.05, demonstrating statistical significance.

## Discussion

This study may be the first to identified latent profiles of digital health readiness among rural Chinese hypertensive patients. Results demonstrated three distinct categories: “low digital health readiness,” “moderate digital health readiness,” and “high digital health readiness,” accounting for 25.2, 54.4, and 20.4% of the sample, respectively. There were significant inter - group differences. The finding that 79.6% of patients exhibited low - to - moderate readiness suggests inadequate digital health readiness in this population, highlighting a critical area for intervention. Using a Health Ecological Model framework, this study analyzed the determinants of the three digital health readiness categories among rural hypertensive patients. Such a systematic analysis enables the formulation of more equitable and effective strategies to improve readiness in this population, especially among vulnerable groups, thus reducing the digital health divide.

### Downstream factors

Personal characteristics, including age, hypertension duration, activities of daily living, and the number of other chronic diseases, were significantly associated with digital health readiness among rural hypertensive patients. Our findings demonstrate that, compared with participants aged ≥70 years, those in the age groups ≤49, 50–59, and 60–69 showed significantly higher odds of being classified into the medium - and high - readiness groups than the low - readiness group. This pattern suggests that age may serve as a critical predictor of digital health readiness among rural hypertensive patients, consistent with the research of Cajita MI et al. (50). Older adults patients may encounter greater difficulty in learning and using smart devices and health applications due to declines in vision and cognitive function or apprehension towards complex technologies. Compared with participants with a short hypertension duration (<5 years), those with a disease course of 5–10 years showed significantly higher odds of belonging to the moderate readiness group (OR = 1.819). Patients with 5–10 years of disease duration may have progressed beyond the initial adaptation phase (51) and may be in the active disease management stage, with clearly defined needs for digital management tools such as health information access and remote monitoring, and concurrently exhibited greater digital health readiness (32). Nonetheless, this readiness remains at the “moderate” level, potentially reflecting persistent barriers in the practical application of digital tools, health information appraisal, or technological adaptation, which may limit advancement to proficient or high - level digital health readiness.

Our results further indicate that participants with no chronic comorbidity were more likely to belong to the high - readiness group compared with those with ≥2 types of chronic comorbidities (OR = 3.092). Individuals managing multiple chronic diseases typically face complex treatment regimens, frequent medical appointments, and polypharmacy, which may deplete the psychological bandwidth necessary for learning, exploring, and adapting to novel digital health technologies. The cross-sectional design precludes determination of whether multimorbidity precedes low readiness or whether digital disengagement exacerbates disease management difficulties. This study also reveals that participants with normal functional status and those with only mild limitations demonstrated higher odds of belonging to the moderate readiness group compared with those with severely restricted activities of daily living (OR = 3.079; OR = 2.576). This aligns with Hungarian research indicating low digital tool utilization among individuals with long - term activity limitations (52). Impaired functional capacity may directly affect the physical ability or willingness to use electronic devices (e.g., smartphones, wearable devices) due to mobility limitations, fatigue, or pain.

These observational patterns generate hypotheses for intervention development. Should future longitudinal or experimental research confirm these associations, implementation of digital health interventions might consider: maintain and strengthen offline channels to ensure basic service accessibility. For example, provide “intergenerational digital assistance” to older adults patients and those with limited functional capacity through village officials, family doctors, and volunteers. Additionally, design personalized and user-friendly digital tools with simplified navigation pathways and voice-interactive services (53, 54) to facilitate long-term disease management.

### Midstream factors

Midstream factors—including regular exercise, awareness of digital health, cardiovascular disease risk perception, number of children, and social support rating—showed indirect, cumulative associations with the digital health readiness of rural hypertensive patients. Our findings demonstrate that participants who engaged in regular exercise were significantly more likely to be classified in the high digital health readiness group compared to the low - readiness group (OR = 2.410). Barnett et al. (55) reported that digital tools optimize exercise behaviors by allowing users to visually track their progress, thereby significantly enhancing self - efficacy. Increased self - efficacy then strengthens users’ reliance on and satisfaction with digital tools, establishing a virtuous cycle (56). Among rural hypertensive patients who do not currently engage in regular physical activity, multi - component behavioral interventions that incorporate motivational enhancement, micro - habit formation, and cognitive restructuring may facilitate the sustained adoption of exercise.

Compared with participants who lacked understanding of digital health, those with some awareness demonstrated significantly increased odds of belonging to the high-readiness group (OR = 3.406). This finding suggests potential value in disseminating digital health knowledge among the public, particularly through prioritized educational and awareness-raising interventions for populations with knowledge deficits. Such targeted efforts may represent actionable entry points for improving population-wide digital health readiness, pending intervention efficacy trials. Our findings indicate that participants with self-rated moderate cardiovascular disease risk were significantly less likely to belong to the high digital health readiness group compared to those with self-rated high risk (OR = 0.427). This finding reveals a potential link between health risk perception and digital health readiness. Individuals’ perception of disease threat is a critical factor motivating health behavior adoption (57, 58). Therefore, targeted training and educational interventions should be considered and to enhance awareness of hypertension and cardiovascular disease, with particular emphasis on improving cardiovascular disease risk perception among rural hypertensive patients.

Our findings indicate that, compared with patients having four or more children, both childless patients and those with two to three children were more likely to be classified into the medium and high digital health readiness groups. An excessive number of children may lead to the diffusion of responsibility (“free - rider effect”), which paradoxically reduces the level of support provided by individual offspring (59). For patients from large families, we tentatively suggest exploring a “family health manager” role—training one willing and capable child to act as a liaison —and testing digital tools with “family sharing” functionality.

Multivariate regression analysis indicated that social support was significantly associated with digital health readiness (OR = 1.069, OR = 1.118), consistent with prior research demonstrating the direct positive effect of social support on health literacy (60). These findings suggest that enhancing social support may be a potential target for interventions seeking to improve digital health readiness.

### Upstream factors

As distal factors, upstream factors (education level, employment status, doctor - recommended use of digital - based blood pressure management devices, and home wireless network coverage) showed indirect associations with rural hypertensive patients’ digital health readiness at a macro level. Compared to participants with a bachelor’s degree or above, rural hypertensive patients with a primary school education or below were significantly less likely to demonstrate high digital health readiness (OR = 0.073). These findings suggest that educational attainment is associated with the acceptance of digital health tools among rural hypertensive patients, where lower educational levels are associated with diminished digital health readiness. Compared with retired participants, employed individuals had significantly higher odds of being classified into both the medium- and high-readiness groups compared to the low-readiness group (OR = 3.621, OR = 3.958). This finding suggests potential value in addressing the specific needs of retired individuals in digital health promotion initiatives. Compared with participants who did not receive physician recommendations, those who received recommendations for digital blood pressure management devices had significantly higher odds of belonging to the medium - readiness (OR = 1.745) and high - readiness groups (OR = 2.762) relative to the low - readiness group. As authoritative sources of health information, physicians’ formal recommendations serve as a critical “social nudge” (61). Implementation strategies might consider testing systematic incorporation of physician recommendations in primary care settings. This study found that participants with home wireless network access demonstrated significantly higher odds of belonging to the moderate digital health readiness group compared with those without wireless network (OR = 2.260). This finding suggests that household internet connectivity is associated with digital health readiness, consistent with prior research (62). Notably, wireless network access was primarily associated with “moderate” rather than “high” readiness. This suggests that while physical connectivity may represents a threshold for initial adoption, it was not sufficient in itself to coincide with high-level readiness in this cross-sectional sample. These observations generate the hypothesis that digital health promotion might benefit from a phased approach: expanding infrastructure coverage to increase user base entry, followed by targeted interventions to enhance readiness.

### Limitations

This study has several limitations. First, regarding generalizability, although our sample was drawn from townships across three distinct regions (Zhuzhou, Liuyang, and Loudi), all sites were confined to Hunan Province, a central-southern Chinese province with specific socioeconomic and cultural characteristics. Consequently, findings may not generalize to rural regions and urban populations in other provinces with differing economic development levels or health infrastructure. Future research employing multi-province, stratified sampling encompassing diverse geographic and socioeconomic contexts—particularly comparative urban–rural designs—would enhance external validity. Second, measurement limitations warrant consideration. Activities of daily living (ADL) were assessed using a single-item self-report measure (“Do you have any difficulties in daily activities?”) rather than a validated multidimensional scale. This approach may lack sensitivity to detect subtle functional impairments and limits comparability with studies employing standardized ADL instruments. Additionally, cardiovascular disease risk perception was measured using a single 0–10 visual analog scale (“What do you think is your risk of cardiovascular disease?”). Single-item measures are prone to low reliability, attenuation bias, and contextual variability in interpretation, potentially underestimating true associations with digital health readiness. Future studies should incorporate validated multi-item scales (e.g., the Perceived Risk Scale or domain-specific risk perception instruments) to improve measurement precision. Third, the cross-sectional design precludes establishing causal relationships or temporal sequencing between associated factors and digital health readiness. The observed associations may reflect reverse causality (e.g., high digital readiness enabling better health management that influences risk perception) or unmeasured confounding. Future studies should employ longitudinal cohort designs with multiple assessment points or natural experiment approaches to disentangle causal pathways and elucidate underlying mechanisms.

## Conclusion

This study employed Latent Profile Analysis (LPA) within a Health Ecological Model framework to comprehensively examine multi-level influences on rural hypertensive patients’ digital health readiness from distal to proximal factors. Analysis identified three distinct latent subgroups with significant differences: Low Digital Health Readiness group, Moderate Digital Health Readiness group, and High Digital Health Readiness group. Influencing factors encompassed age, hypertension duration, number of chronic comorbidities, activities of daily living, regular exercise, digital health awareness, cardiovascular disease risk perception, number of children, social support rating, educational level, employment status, doctor - recommended use of digital - based blood pressure management devices, and home wireless network coverage. These findings underscore that rural primary care clinicians and health service institutions must understand the heterogeneous characteristics of older populations and implement targeted interventions to enhance digital health readiness, thereby improving health outcomes and reducing health disparities.

## Data Availability

The raw data supporting the conclusions of this article will be made available by the authors, without undue reservation.
